# Ecological factors and childhood eating behaviours at 5 years of age: findings from the ROLO longitudinal birth cohort study

**DOI:** 10.1186/s12887-022-03423-x

**Published:** 2022-06-27

**Authors:** Anna Delahunt, Marie C. Conway, Eileen C. O’Brien, Aisling A. Geraghty, Linda M. O’Keeffe, Sharleen L. O’Reilly, Ciara M. McDonnell, Patricia M. Kearney, John Mehegan, Fionnuala M. McAuliffe

**Affiliations:** 1 UCD Perinatal Research Centre, School of Medicine, University College Dublin, National Maternity Hospital, Dublin 2, Ireland; 2 School of Biological and Health Sciences, Technological University Dublin, Dublin, Ireland; 3 School of Public Health, College of Medicine and Health, University College Cork, Co Cork, Ireland; 4 School of Agriculture and Food Science, University College Dublin, Dublin 4, Ireland; 5 Department of Paediatric Endocrinology and Diabetes, Children’s Health Ireland Temple St and Tallaght, Dublin, Ireland

**Keywords:** Childhood, Eating behaviours, Ecological, Socio-economic status, Childcare, Screen time, Overweight, Obesity

## Abstract

**Background:**

Individual differences in children eating behaviours have been linked with childhood overweight and obesity. The determinants of childhood eating behaviours are influenced by a complex combination of hereditary and ecological factors. This study examines if key ecological predictors of childhood overweight; maternal socio-economic status (SES), children’s screen time, and childcare arrangements, are associated with eating behaviours in children aged 5-years-old**.**

**Methods:**

This is secondary, cross-sectional analysis of the ROLO (Randomized COntrol Trial of LOw glycemic diet in pregnancy) study, using data from the 5-year follow-up (*n* = 306). Weight, height, and body mass index (BMI) were obtained from mothers and children at the 5-year follow-up. Children’s BMI z-scores were calculated. SES was determined using maternal education level and neighborhood deprivation score. Information on children’s screen time and childcare arrangements were collected using lifestyle questionnaires. Children’s eating behaviours were measured using the Children’s Eating Behaviour Questionnaire (CEBQ). Multiple linear regression, adjusted for potential confounders, assessed associations between maternal SES, screen time and children’s eating behaviours. One-way ANOVA, independent sample t-tests and Spearman’s correlation examined childcare exposure and children’s eating behaviour.

**Results:**

Mothers in the lowest SES group had higher BMI and were younger than those in the highest SES group (*p* =  < 0.001, *p* = 0.03 respectively). In adjusted analysis, the lowest SES group was associated with a 0.463-point higher mean score for ‘Desire to Drink’ (95% CI = 0.054,0.870, *p* = 0.027) and higher ‘Slowness to Eat’ (B = 0.388, 95% CI = 0.044,0.733, *p* = 0.027) when compared with the highest SES group. Screen time (hours) was associated with higher ‘Food Fussiness’ (B = 0.032, 95% CI = 0.014,0.051, *p* = 0.001). Those who attended childcare had higher scores for ‘Desire to Drink’(*p* = 0.046). No relationship was observed between longer duration (years) spent in childcare and eating behaviours.

**Conclusions:**

In this cohort, the ecological factors examined had an influence on children’s eating behaviours aged 5-years-old. Our results illustrate the complexity of the relationship between the child’s environment, eating behaviour and children’s body composition. Being aware of the ecological factors that impact the development of eating behaviours, in the pre-school years is vital to promote optimal childhood appetitive traits, thus reducing the risk of issues with excess adiposity long-term.

**Supplementary Information:**

The online version contains supplementary material available at 10.1186/s12887-022-03423-x.

## Introduction

**Fig. 1:**
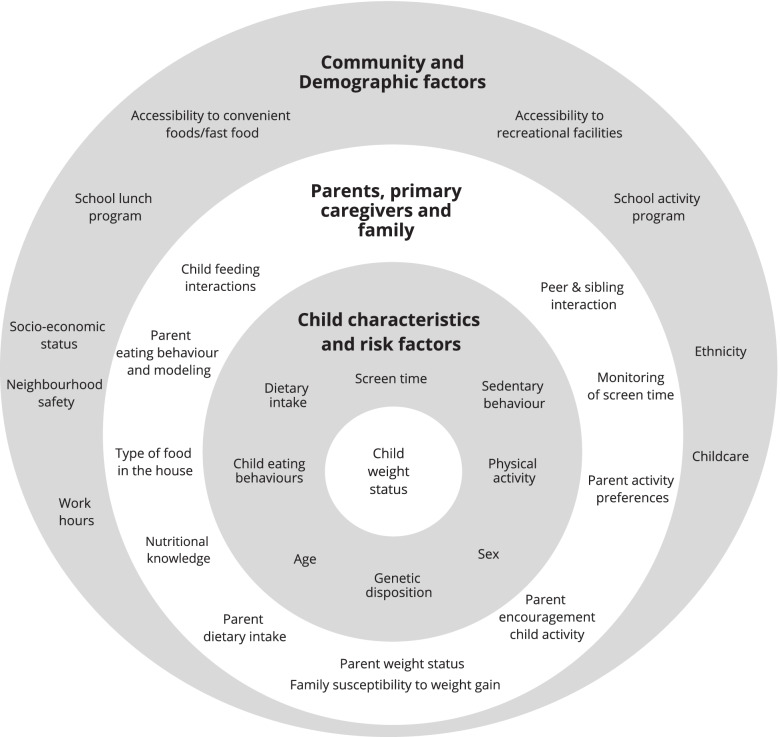
Ecological model of predictors of childhood overweight. Adapted from Davidson and Birch (refererence 1) and reprinted with permssion from Obesity Reviews. (Ref 1; Davidson KK, Birch LL. Childhood overweight: a contextual model and recommendations for future research. Obes Rev. 2001;2(3) 159-71)

Childhood obesity is a growing problem globally and its etiology is complex. It is because of this complexity that prevention is key, ensuring children get the best start in life. The ecological model of health can be used to map risk factors and better understand their relationship to childhood obesity [1, 2]. This model shows the breadth of etiological factors involved in childhood overweight and obesity, including the impact of parents and family, community demographics and socio-economic status (SES), food intake, physical and sedentary activity and the child’s own personal characteristics (Fig. 1). Many of these same factors are influential in determining how a child’s eating behaviours will evolve [3].

Childhood food approach eating behaviours such as food responsiveness and emotional overeating, are most commonly associated with increased weight [4–6]. Children with food responsive eating behaviour (heightened responsiveness to food stimuli, regardless of hunger), have higher meal frequency [7] and display more snacking behaviour [8, 9]. The relationship between weight status and food avoidant eating behaviour such as fussy eating behaviour is more complex. Food fussiness is associated with both greater risk of being underweight [6, 10] and being overweight [11]. Children with fussy eating commonly have limited variety in their diet [12] particularly fruit and vegetables, but may overeat other food groups such as carbohydrates and fats [13]. Understanding the robustness of the relationships between aspects of a child’s ecological framework and a child’s eating behaviour is important for future behavioural intervention strategies.

Lower socio-economic status (SES) is a marker for an obesogenic environment which can impact eating behaviours [14]. Recent data from Ireland and the UK have reported that children from the most deprived backgrounds are more likely to have overweight or obesity than those from more advantaged backgrounds [15, 16]. It is postulated that those from lower SES households may be vulnerable to appetite irregularities due to the combined effect of lower breastfeeding rates and the adversity and stress associated with being disadvantaged [17]. Eating behaviours such as satiety responsiveness, food preference, and selective attention towards food have been shown to be impacted by being from a disadvantaged household [18, 19]. SES is also known to influence type and quality of dietary intake in childhood [20]. A longitudinal study of 8–12-year-old children and their parents, demonstrated that children of mothers with higher educational attainment ate more fruit, vegetables and included daily breakfast more often [21]. The relationship between SES and food avoidant eating behaviours, such as fussy eating, is less clear, with contrasting results as to how SES relates to eating style [10, 22].

Family environment and particularly parental SES has been shown to influence screen time exposure in young children [23]. Furthermore, excessive screen time has been linked with unhealthy eating behaviours in children aged 5–6 years old and young adolescents (11–12 years old), such as increased snacking on energy dense foods and low fruit and vegetable intake [24, 25]. Evidence relating to the impact of screen time on eating behaviours, such as fussy eating, is lacking.

Societal changes over the past three decades have led to large proportions of children spending time in childcare in their preschool years. International data reports that approximately 50% of 3-to-6-year old’s and 25% of infants under 3 years old are exposed to some out of home childcare [26, 27]. Childcare attendance in both formal (preschool/creche-based) and informal (relative/family) settings have been associated with childhood overweight and obesity [27–29]. However, findings are inconsistent and multifaceted [30, 31]. Childcare classified as ‘Informal’ has been linked to early introduction to solid food, less physical activity and excess adiposity [30]. Children can spend a considerable amount of time in childcare thus making it a key location for the establishment of eating behaviours. The role of peers in the preschool setting has been found to be influential in shaping dietary patterns and the physical activity levels of their fellow counterparts [32]. In addition, other factors such as the attitudes and behaviours of the childcare providers towards food and their responsiveness to the child’s eating style have to be considered.

Overall, there is a paucity of literature in relation to how the ecological factors in which a child is embedded within influence eating behaviours, and consequently weight status. To address this gap, the current study’s primary aim was to investigate three components of the ecological model of predictors of childhood overweight; namely maternal SES, child screen time exposure and childcare arrangements, and their associations with children’s eating behaviours in children aged 5 years old. To further elucidate the potential influence of ecological factors on child eating behaviours, a secondary aim explored maternal characteristics and child early feeding across SES groups.

## Methods

### Study details

**Fig. 2:**
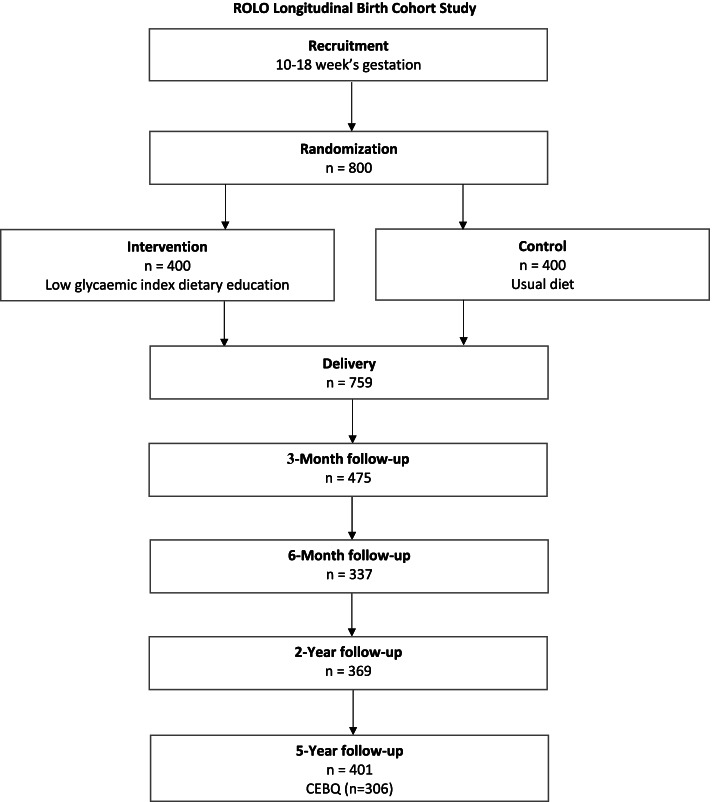
Flow chart of the ROLO longitudinal birth cohort study. ROLO; Randomized control trial of low glycaemic index diet in pregnancy

The ROLO (randomized control trial of low glycemic index diet in pregnancy) study is an ongoing longitudinal birth cohort. The primary study took place in the National Maternity Hospital, Dublin, Ireland from 2007–2011 and focused on a low glycemic index diet during pregnancy, with the aim of preventing the recurrence of fetal macrosomia [33]. Findings from the primary ROLO study have been published elsewhere [33]. The mothers and infants from this study (*n* = 759) have been followed-up as part of the ROLO longitudinal birth cohort study. Participants have been followed-up at numerous time points including at 3 and 6 months, 2 years and when children were aged 5 years old for the ROLO Kids study. ROLO Kids consisted of 401 mother–child dyads and will be the focus for this cross-sectional analysis. Of the 401 children that returned at the 5-year-old follow-up, complete data on child eating behaviours at 5 years old was available for 306 participants, resulting in a final sample of 306 mother–child dyads. A study flow chart detailing the progression of the ROLO study and participant numbers at each follow-up can be seen in Fig. 2. Ethical approval was granted by Our Lady’s Children’s Hospital, Dublin (OLCHC) and the National Maternity Hospital (NMH), Dublin Ethics Committees (Ethics reference number: GEN/279/12).

### Child anthropometry at 5-year follow-up

Weight was measured to the nearest 0.1 kg, using a calibrated stand-on digital weighing scale (SECA 813, Hamburg, Germany). Standing height was measured, without shoes, with head aligned in the Frankfort plane, using a free-standing stadiometer (SECA 217, Hamburg, Germany) and measurements were recorded to the nearest 0.1 cm. Body mass index (BMI) was calculated as kilogram per meter squared (kg/m^2^). Children’s BMI scores were converted to standardized z-scores according to the 1990 UK age- and sex-specific reference data using Excel LMS Growth macro [34, 35]. BMI z-scores were categorized using the World Health Organization criteria for children aged 5 to 19 years [36]. All measurements were taken at the 5-year-old follow-up study day as per study protocol and were carried out by trained researchers.

### Children's eating behaviour at 5-year follow-up

Children’s eating behaviours were measured at 5 years old using the Children’s Eating Behaviour Questionnaire (CEBQ) [37]. The CEBQ is a 35-item, parent reported, validated psychometric tool, developed to capture individual differences in eating styles that may contribute to both underweight and overweight in children [37]. The CEBQ has been validated in a British cohort of children aged 4–5 years old as an accurate measurement of child eating behaviours, displaying good internal consistency and good test–retest reliability [38]. To date the CEBQ has not been validated in an Irish cohort. The CEBQ has eight subscales, four that measure food approach eating behaviours and four that measure food avoidant eating behaviours. The food approach eating behaviour subscales include: ‘Food Responsiveness’ (5 items), measuring heightened responsiveness to external food cues, ‘Enjoyment of Food’ (4 items) measuring a general liking for eating, ‘Emotional Overeating’ (4 items) measuring food intake in response to negative emotions such as anger or anxiety and ‘Desire to Drink’ (3 items) which measures an increased desire to drink, particularly of sugary drinks. The food avoidant behaviour subscales include: ‘Satiety Responsiveness’ (5 items) which measures the degree of self-regulation of food consumed by the child, based on the sensation of feeling full, ‘Emotional Undereating’ (4 items) measuring a decrease in food intake due to negative emotions such as anger or anxiety, ‘Slowness in Eating’ (4 items) measuring the speed at which a child eats, with slowness representing disinterest in food, and ‘Food Fussiness’ (6 items) which measures, a lack of interest in food, or for trying new foods. Responses to the CEBQ statements are scored on a 5-point Likert scale (1 = Never, 5 = Always) with five statements within the CEBQ being reverse scored due to opposite phrasing. To determine the score for each subscale, the items within the subscale were summed and its mean calculated by dividing by the number of items within the subscale. A higher score indicates the child is more likely to express this eating behaviour.

### Assessment of screen time

Screen time was assessed at 5 years old, using the Children’s Leisure Activities Study Survey (CLASS) questionnaire, which is validated for children aged 5–6 years old [39]. Screen time was defined as a combination of three activities; watching television/video games, computer games, and internet. Mothers were asked to report on the amount of screen time minutes for a typical week (Monday to Friday) and a typical weekend (Saturday and Sunday). Screen time was calculated in minutes per week, and analyzed in hours per week.

### Parenting, feeding and parent characteristics

Maternal weight was measured at the same visit as her child’s and used the same methodology. Maternal education was self-reported and recorded at recruitment for the ROLO pregnancy study. Mothers selected one of the following categories; ‘no schooling’; ‘primary education only’, ‘some secondary level education’, ‘complete secondary level education’, ‘some third level education (certificate/diploma)’ or ‘complete third level education (higher-level degree)’.

Mothers reported breastfeeding duration retrospectively, at 6 months, 2 years and 5 years postnatally. At each timepoint, mothers reported if they had breastfed or not and for how long. At the 2 year and 5-year follow-up mothers reported the age (weeks) their infant had commenced solids. A variable was created to indicate if their child had started solids as per national recommendations [40], that is; that complementary foods are not introduced before four months (17 weeks), but should not be delayed beyond six months (26 weeks).

### Community and demographic factors

The Pobal Haase & Pratschke Deprivation Index (HP Index) was used to allocate a deprivation score as per the participants address or small area [41]. Participants addresses were obtained during the ROLO pregnancy study (2007–2011). The HP Index is derived from data from the ‘2011 Census of Population in Ireland’, and provides information on a combination of three dimensions of relative affluence and deprivation, specifically demographic profile, social class composition and labor market supply and demand. The HP Index data is normally distributed; with the mean fixed at 0. Thus, a negative score (below 0) is classified within the disadvantaged categories and a positive score (above 0) being grouped within the advantaged categories. SES was determined by creating a composite variable, using maternal education level and Pobal HP Index [41]. Four SES categories were created based on level of education and neighborhood deprivation index: ‘Third level and Advantaged’, ‘Third level and Disadvantaged’, ‘Less than third level and Advantaged’ and ‘Less than third level and Disadvantaged’.

Information on childcare was obtained from maternal reported lifestyle questionnaires at the 5-year follow-up. In the current analysis, childcare attendance was defined as non-parental care, which may have been in the home or outside the home. Mothers were asked an open-ended question about whether their child attended childcare or not. Categories provided for mothers to describe their childcare arrangements included; crèche or preschool (formal), childminder in the home, childminder outside the home, relative in the home, relative outside the home (informal) or other (asked to specify). Duration of childcare attendance was calculated by subtracting the age of childcare commencement from the age of the child at the 5-year examination. The childcare questionnaire asked whether food was provided, and this was classified into five groups; ‘Yes, all meals’, ‘Yes, main meals only’, ‘Yes, snacks only’, ‘No meals’ or ‘Don’t know’.

### Statistical analysis

Continuous data were tested for normality using the Kolmogorov–Smirnov test and visual inspection of histograms. Normally distributed variables were reported as mean and standard deviation (SD). Non-parametric variables were reported as median and interquartile range (IQR). Eating behavior variables were normally distributed and parametric tests were used. One-way ANOVA with post-hoc Tukey’s tests were used to explore crude mean differences in maternal and child characteristics across SES groups. Maternal BMI was non-normally distributed; therefore, Kruskal–Wallis and Mann Whitney U tests were used to examine these characteristics across SES. Chi-squared test for independence was completed to examine breast feeding exposure across SES. Unadjusted and adjusted linear regression analysis were performed to examine associations between maternal SES and children’s eating behaviours at age 5. For this analysis dummy variables were created, with the largest category ‘Third level and Advantaged’ used as the reference variable. Conditions of independence, linearity, normality and homoscedasticity were tested and met prior to all linear regression analyses. Cronbach α was performed on each subscale of the CEBQ to assess the internal consistency of each subscale in our cohort.

Total screen time exposure was analyzed in hours per week. Adjusted and non-adjusted linear regression were completed to examine associations between children’s screen time and eating behaviours.

Independent sample t-tests examined differences in eating behaviours and child’s body composition for children who had attended childcare and those who did not. Meals provided in childcare were recategorized into two groups, ‘food provided’ and ‘no food provided’. Independent sample t-tests were completed to examine differences between these two groups. Spearman’s correlations were completed to assess relationships between duration of time spent in childcare and children’s eating behaviours. Type of childcare attendance was stratified into 3 groups – ‘formal’ (creche/preschool), ‘informal’ (nanny or relative inside or outside the home) and ‘mixed’ – combination of both. One-way ANOVA assessed differences in eating behaviour and children’s body composition across type of childcare.

All multiple regression models were adjusted for maternal BMI at the 5-year follow-up, child breastfeeding exposure, whether the child met national guidelines for age starting solids or not, child age at 5-year follow up, child sex, and original RCT allocation group. Maternal SES was also included as a confounder in the screen time regression models. A *p*-value of < 0.05 was considered statistically significant. Statistical analyses were completed using IBM Statistical Package for Social Sciences (SPSS) for Windows, version 24.0. Armonk, NY: IBM, Corp.

## Results

The study group characteristics are presented in Table 1. At the 5-year follow-up 61.8% of women had completed third level education or above and lived in an advantaged area. Children’s mean age at follow-up was 5.1 years, with 47% males and 53% females. Of these, 24% had a BMI z-score in the overweight or obese range. Median screen time exposure for children was 11.0 h per week. At the 5-year follow-up 89.5% of children had attended childcare, with median childcare exposure 4.1 years (Table 1).

**Table 1:** General characteristics of the ROLO mother and child dyads at the 5-year follow-up

	**n (%)**	**Mean (Median)**	**SD (IQR)**
**Maternal characteristics**			
Mothers age at 5-year follow-up	306	38.44	3.90
Maternal BMI at 5-year follow-up (kg/m^2^)^a^	292	(25.87)	(22.77, 28.23)
**Maternal BMI category**^b^			
Underweight (< 18.5 kg/m^2^) n (%)	4 (1.4)		
Healthy (18.5–24.9 kg/m^2^) n (%)	143 (49.0)	-	-
Overweight (25–29.9 kg/m^2^) n (%)	97 (33.2)	-	-
Obesity (≥ 30 kg/m^2^) n (%)	48 (16.4)	-	-
Completed third level education or above n (%)	173 (61.8)	-	-
Third level and Advantaged n (%)	145 (51.8)	-	-
Third level and Disadvantaged n (%)	30 (10.7)	-	-
Less than third level and advantaged n (%)	77 (27.6)	-	-
Less than third level and disadvantaged n (%)	37 (10.0)	-	-
**Child characteristics**			
Child age (years)	306	5.18	0.15
Child sex (male), n (%)	141 (46.5)		
Child BMI z-score	293	0.40	0.87
Healthy weight (> -2 and < + 1SD) n (%)^c^	223 (76.1)	-	-
Overweight (> + 1 SD) n (%)^c^	58 (19.8)	-	-
Obese (> + 2 SD) n (%)^c^	12 (4.1)	-	-
**Child early feeding and eating behaviours**			
Some breastfeeding exposure n (%)	208 (68.0)	-	-
Age of introduction of solids (weeks)	298	23.11	6.70
Met recommendations for timing of introduction to solids n (%)	255 (83.3)	-	-
Food Responsiveness (FR) (5 items)	306	2.49	0.82
Emotional Overeating (EOE) (4 items)	306	1.65	0.52
Enjoyment of Food (EF) (4 items)	306	3.73	0.76
Desire to Drink (DD) (3 items)	306	2.67	0.93
Satiety Responsiveness (SR) (5 items)	306	3.06	0.66
Slowness Eating (SE) (4 items)	306	3.04	0.78
Emotional Undereating (EUE) (4 items)	306	2.70	0.86
Food Fussiness (FF) (6 items)	306	3.08	0.98
**Children’s screen time**			
Screen time per week^a^	226	(11.00)	(7.88,16.50)
**Childcare**			
Attended childcare n (%)	274 (89.5)	-	-
Time spent in childcare (years)^a^	232	(4.14)	(2.31,4.44)
Formal childcare (Creche/Preschool) n (%)	148 (59.0)	-	-
Informal childcare (nanny or relative in or out of home) n (%)	77 (30.6)	-	-
Both formal and informal childcare n (%)	26 (10.4)	-	-
Food provided in childcare n (%)	153 (54.8)	-	-

Results presented as mean and standard deviation (SD) for normally distributed variables and.^a^Median and interquartile range (25^th^-75.^th^ percentile) for non-normally distributed variables; Categorical data presented as n (%); ^b^WHO BMI classification; ^c^WHO cut-offs for BMI z-scores for children aged 5–19 years old; Child eating behaviours assessed using CEBQ [37]; Food approach eating behaviours: degree to which a child has a more avid appetite and greater interest in food (includes FR, EOE,EF,DD), Food avoidant eating behaviours: degree to which a child has a smaller appetite and is less interested in food (includes SR, SE, EUE, FF). Mean and SD of CEBQ subscales are derived from the sum of subscale divided by number of items within the subscale

*Abbreviations*: *ROLO* Randomized control trial of low glycaemic index diet in pregnancy, *SES* Socio-economic status

A total of 306 mothers completed a CEBQ for their child with internal reliability coefficients (Cronbach’s α) ranging from 0.695 to 0.928, thus all questions were included in the analysis. The Cronbach α value for each factor is as follows: ‘Food Responsiveness’ (5 items) 0.822, ‘Emotional Overeating’ (4 items) 0.758, ‘Enjoyment of Food’ (4 items), 0.890, ‘Desire to Drink’ (3 items), 0.864, ‘Satiety Responsiveness’ (5 items), 0.779, ‘Slowness Eating’ (4 items) 0.792, ‘Emotional Undereating’ (4 items) 0.695, and ‘Food Fussiness’ (6 items), 0.928. Mean scores and standard deviations (SD) for children’s eating behaviours are described in Table 1.

Differences between the ‘Third level and Advantaged’ and ‘Less than third level and Disadvantaged’ SES groups were evident for maternal age (Mean 39.4 ± 3.21 versus 35.82 ± 5.24, *p* = 0.001) (Additional file 1, Table 1). Maternal BMI at 5-year follow-up was lower in the ‘Third level and Advantaged’ group compared with ‘Less than third level and Advantaged’ and ‘Less than third level and Disadvantaged’ (Median 24.35, IQR = 22.42, 26.85 versus Median 25.58, IQR = 23.78,29.82; Median 25.04, IQR = 23.73, 29.96, *p* = 0.03 respectively) (Additional file 1, Table 1). ‘Desire to Drink’ scores were lower for children of mothers in the ‘Third level and Advantaged’ SES category compared with the ‘Less than third level and Disadvantaged’ SES category (Mean 2.51 ± 0.81 versus Mean 3.01 ± 1.08, *p* = 0.01) (Additional file Table 1). A chi squared test for independence indicated a significant difference across SES groups between those who had breastfed or not (χ^2^(3, *n* = 244) = 48.72, *p* = 0.001), with lower breastfeeding exposure in the ‘Less than third level and Disadvantaged’ group compared with the ‘Third level and Advantaged’ group (Additional file 2, Table 2).

In adjusted analysis, ‘Less than third level and Disadvantaged’ and ‘Less than third level and Advantaged’ were associated with higher ‘Desire to Drink’ (B = 0.462, 95% CI = 0.054, 0.870, *p* = 0.027; B = 0.297, 95% CI = 0.026,0.568, *p* = 0.032 respectively) when compared to ‘Third level and Advantaged’ (Table 2). ‘Less than third level and Disadvantaged’ was associated with higher ‘Slowness to Eat’ (B = 0.388, 95% CI = 0.044, 0.733, *p* = 0.027) when compared with ‘Third level and Advantaged’ (Table 2).

**Table 2:** Association between maternal socio-economic status at time of birth and children’s eating behaviours at aged 5 years old

		**Education-deprivation category as a marker of SES**			
		**Third level and Advantaged**	**Third level and Disadvantaged**	**Less than third level and Advantaged**	**Less than third level and Disadvantaged**
**Food Responsiveness** **(FR)**	**B (95% CI)**	*Ref*	0.240 (-0.170,0.587)	0.251 (0.007,0.496)	0.095 (-0.273,0.463)
	***P*-value**		0.175	0.044	0.689
	**Adj R**^**2**^		-0.001		
**Emotional Overeating** **(EOE)**	**B (95% CI)**	*Ref*	0.059 (-0.161,0.279)	-0.038 (-0.649,0.574)	-0.034 (-0.267,0.200)
	***P*-value**		0.597	0.833	0.776
	**Adj R**^**2**^		-0.018		
**Enjoyment of Food** **(EF)**	**B (95% CI)**	*Ref*	0.020 (-0.303,0.344)	-0.121 (-0.349 0.107)	-0.060 (-0.403,0.283)
	***P*-value**		0.902	0.299	0.731
	**Adj R**^**2**^		-0.017		
**Desire to Drink** **(DD)**	**B (95% CI)**	*Ref*	0.210 (-0.175,0.594)	0.297 (0.026,0.568)	0.462 (0.054,0.870)
	***P*-value**		0.285	0.032	0.027
	**Adj R**^**2**^		0.021		
**Satiety Responsiveness** **(SR)**	**B (95% CI)**	*Ref*	-0.231 (-0.509,0.048)	0.076 (-0.121,0.272)	0.075 (-0.220,0.371)
	***P*-value**		0.104	0.448	0.617
	**Adj R**^**2**^		0.006		
**Slowness Eating** **(SE)**	**B (95% CI)**	*Ref*	-0.186 (-0.511,0.139)	0.138 (-0.091,0.367)	0.388 (0.044,0.733)
	***P*-value**		0.261	0.236	0.027
	**Adj R**^**2**^		0.018		
**Emotional Undereating** **(EUE)**	**B (95% CI)**	*Ref*	0.129 (-0.234,0.491)	-0.016 (-0.272, 0.239)	0.091(-0294, 0.475)
	***P*-value**		0.486	0.901	0.643
	**Adj R**^**2**^		0.001		
**Food Fussiness** **(FF)**	**B (95% CI)**	*Ref*	0.104 (-0.303, 0.510)	-0.233 (-0.054, 0.520)	-0.405 (-0.836, 0.027)
	***P*-value**		0.616	0.111	0.066
	**Adj R**^**2**^		0.043		

Third level and Advantaged was used as reference to which other SES categories (Third level and Disadvantaged; Less than third level and Advantaged; Less than third level and Disadvantaged) were compared. *CI* Confidence interval, *Adj* Adjusted; All multiple regression models were adjusted for maternal BMI at 5-year follow-up, child breastfed ever, whether child met national guidelines for age of solids introduction or not, child age at 5-year follow-up, child sex, original RCT allocation group; Food approach eating behaviours: degree to which a child has a more avid appetite and greater interest in food (includes FR, EOE,EF,DD), Food avoidant eating behaviours: degree to which a child has a smaller appetite and is less interested in food (includes SR, SE, EUE, FF). Statistically significant (*p*- value < 0.05)

In adjusted regression models, child screen time exposure (hours) was associated with higher ‘Food Fussiness’ (B = 0.032, 95% CI = 0.014, 0.051, *p* = 0.001) (Table 3). No other associations were observed between screen time exposure and any other eating behaviour.

**Table 3:** Association between children’s screen time exposure and children’s eating behaviour at 5 years old

	**Screen time exposure (hours)**				
	**B**	**95% CI**		***P*-value**	**Adj R**^**2**^
		**Lower**	**Upper**		
Food Responsiveness (FR)	-0.008	-0.007	0.024	0.296	-0.011
Emotional Overeating (EOE)	-0.001	-0.011	0.009	0.869	-0.031
Enjoyment of Food (EF)	-0.008	-0.022	0.007	0.310	-0.016
Desire to Drink (DD)	0.009	-0.008	0.025	0.321	0.036
Satiety Responsiveness (SR)	0.012	0.000	0.024	0.059	0.043
Slowness Eating (SE)	0.006	-0.022	0.020	0.398	0.048
Emotional Undereating (EUE)	-0.006	-0.022	0.010	0.471	0.018
Food Fussiness (FF)	0.032	0.014	0.051	0.001	0.038

Multiple linear regression; *CI* Confidence Interval, *Adj* Adjusted; All multiple regression models adjusted for maternal BMI at 5-year follow-up, maternal SES, child breastfed ever, whether child met national guidelines for age of solids introduction, child age at 5-year follow-up, child sex, original RCT allocation group; Food approach eating behaviours: degree to which a child has a more avid appetite and greater interest in food (includes FR, EOE,EF,DD), Food avoidant eating behaviours: degree to which a child has a smaller appetite and is less interested in food (includes SR, SE, EUE, FF). Statistically significant (*p*- value < 0.05)

Of the children who attended childcare, 59% availed of formal childcare, 31.6% of informal childcare, and 10.3% attended a combination of both formal and informal (Table 1). Children who had attended childcare had higher mean scores for ‘Desire to Drink’ than those who had never attended childcare (Mean 2.70 ± 0.94 versus Mean 2.42 ± 0.72, *p* = 0.046) (Table 4). No differences were seen in child eating behaviours across type of childcare. In correlation coefficient analysis, no relationships were observed between duration of time spent in childcare and childhood eating behaviours (Additional file 3, Table 3). In relation to provision of food at childcare, 57% of children received some food in childcare, 40.6% received no food, 2.4% did not know (Table 1). No differences were observed in eating behaviours between those who received food compared to those who did not.

**Table 4:** Children’s eating behaviours across childcare attendance in 5 years old children

	**Attended childcare**			**Did not attend childcare**			
	**n**	**Mean**	**SD**	**n**	**Mean**	**SD**	***P*-value**
Food Responsiveness (FR)	274	2.51	0.82	32	2.29	0.84	0.168
Emotional Overeating (EOE)	274	1.65	0.44	32	1.63	0.44	0.834
Enjoyment of Food (EF)	274	3.74	0.75	32	3.65	0.81	0.537
Desire to Drink (DD)	274	2.70	0.94	32	2.42	0.72	0.046
Satiety Responsiveness (SR)	274	3.07	0.66	32	2.96	0.65	0.385
Slowness Eating (SE)	274	3.05	0.76	32	3.02	0.91	0.896
Emotional Undereating (EUE)	274	2.70	0.87	32	2.61	0.85	0.574
Food Fussiness (FF)	274	3.06	0.99	32	3.26	0.96	0.287

*P*- value determined from Independent sample t-tests for differences between groups. Food approach eating behaviours: degree to which a child has a more avid appetite and greater interest in food (includes FR, EOE, EF, DD), Food avoidant eating behaviours: degree to which a child has a smaller appetite and is less interested in food (includes SR, SE, EUE, FF). Statistically significant (*p* value < 0.05)

## Discussion

The present study explored three predictors of childhood overweight, which feature in the ecological model; SES, screen time exposure and childcare arrangements, and their associations with children’s eating behaviours at 5 years old. We also explored maternal characteristics, child early feeding and eating behaviours across SES groups. Our findings indicate that children of mothers with lower than third level education and living in a disadvantaged area exhibited higher ‘Desire to Drink’ and ‘Slowness to Eat’ scores when compared to children of mothers who had completed third level or above education and lived in an advantaged area. Screen time exposure was positively associated with ‘Food Fussiness’. At 5 years old, attending childcare was positively associated with ‘Desire to Drink’.

Apart from ‘Desire to Drink’, no other food approach eating behaviours were associated with SES in this cohort. We had expected to observe associations between SES and other food approach appetitive traits, such as ‘Food Responsiveness’ and ‘Enjoyment of Food’ in view of an already established connection between these eating behaviours and childhood overweight and obesity [42], and the higher prevalence of overweight and obesity in disadvantaged backgrounds [15, 16]. However, it is important to highlight recent evidence showing a strong genetic component in the development of these particular appetitive traits [43, 44]. Twin studies have demonstrated that ‘Food Responsiveness’ is highly heritable [45, 46] however, it has also been shown that the behavioural manifestation of heightened responsiveness to food cues is also dependent on the contribution of environmental factors [47, 48]. ‘Less than third level and Disadvantaged’ was positively associated with a 1.30-point increase in mean score for ‘Desire to Drink’ compared to ‘Third level and Advantaged’. Similar results have been shown in previous studies in low-income families [49, 50]. In the Generation R study, lower SES at 5 years was associated with higher ‘Desire to Drink’, higher ‘Food Responsiveness’ and higher ‘Emotional Overeating’[49]. Furthermore, a study from the UK, observed associations between higher ‘Desire to Drink’ and increased intake of sugar sweetened beverages (SSB) [51]. Dietary patterns that include high intakes of SSB have been linked with childhood obesity [52, 53], increased food intake [51] and a positive energy balance [54]. However, in the CEBQ, the construct of ‘Desire to Drink’ does not provide information on the actual type of beverage the child was likely to consume or request. Further research exploring the relationship between dietary patterns across SES groups and ‘Desire to drink’ is warranted.

Our cohort reflected current national data of lower rates of breastfeeding in lower SES groups [55]. In previous research from the ROLO cohort, an association was observed between lower breastfeeding duration and higher scores for ‘Desire to Drink’ [56]. In the current study, even following adjustment for breastfeeding exposure, a positive association between ‘Less than third level and Disadvantaged’ and ‘Desire to Drink’ remained, suggesting that the association was not influenced by breastfeeding exposure.

The only associations observed between SES and the food avoidant eating behaviours was with ‘Less than third level and Disadvantaged’ and higher mean scores for ‘Slowness Eating’. ‘Slowness Eating’ represents a small appetite and disinterest in food. Evidence shows that children from lower SES households will be exposed to a more ‘obesogenic’ environment with greater access to unhealthy foods, less structured meal times and less parental responsive feeding practices [57, 58]. Research also suggests that lower SES households have higher intakes of SSB [59] and children who drink excessively often do have poorer appetites, as the volume of fluid intake can displace hunger. Previous research has found that fussy eating is more common in lower SES households [10, 60]. However contrasting results have also been demonstrated, showing associations between lower SES and lower scores for food fussiness and higher scores for ‘Food Responsiveness’ ‘Enjoyment of Food’, with no associations observed between SES and ‘Slowness eating [49].

In recent years an environmental factor that is inevitably part of the lives of both adults and children is exposure to screen time. It is recommended that children aged 2–5 years old spend no more than 1 h per day exposed to screens [61, 62]. Our cohort had an average of 1.8 h of screen time per day. Excessive screen time is associated with unhealthy eating behaviours, such as increased snacking on energy dense foods and reduced intake of fruit and vegetables [24, 25]. In the current analysis, none of the food approach eating behaviours were associated with screen time exposure, however, screen time exposure was positively associated with ‘Food Fussiness’. As screen time has been associated with increased snacking or grazing behaviour this may consequentially predispose the child to a poorer appetite at meal times, reduced satiety cues and a less structured meal time environment. A structured meal time environment for children has been proposed as an important strategy for reducing fussy eating and helping a child to recognize their hunger cues. Structured meal times include the provision of a routine, reduced distractions at the meal and having the family present for meals [63]. However, this association potentially could be bi-directional, as children with fussy eating and/or sensory issues may avoid sitting at the table with family for meals and screen time may be used as a distraction technique to help encourage eating. In a previous study of preschool children, parents of children who demonstrated fussy eating behaviour reported that their child had too much screen time [64]. To our knowledge, the association between screen time and fussy eating in children has not been previously demonstrated. However, further research is warranted which includes data on dietary patterns to help fully understand this association between screen time exposure and fussy eating behaviour.

Currently, research is limited on how childcare impacts children’s eating behaviours. Research from the child development field indicates that the quality of childcare is an important determinant of positive or negative childhood developmental outcomes, particularly for those from disadvantaged backgrounds [65]. We observed higher scores for ‘Desire to Drink’ for those who attended childcare versus those who did not. However, when childcare was categorized into formal/informal or a combination of both, no differences were observed. As previously discussed, research on preschool children has demonstrated that the construct of ‘Desire to Drink’ was related to an increased desire for sugar sweetened beverages, and that this was not driven by thirst [51].

A positive finding in our cohort was that no associations were observed between duration of exposure to childcare and any of the food avoidant eating behaviours. To date, limited research is available on the impact of childcare on food avoidant eating behaviours and whether fussy eating perception differs between a child's parent and their childcare provider. In a small study of 3–5-year-old children, video observation of eating patterns at home and in childcare demonstrated that children exhibited more fussy eating behaviour at home than in childcare [66]. The influence of peers may play a role in diminishing food avoidant eating behaviour in the childcare setting, as children tend to imitate and learn from their peers [32]. Longitudinal data is required to examine how children’s peers within the childcare setting may influence eating behaviours over time.

Strengths of this study include the use of a validated questionnaire to measure childhood eating behaviours. Combining maternal educational level and neighborhood deprivation (HP Index) to form an education-deprivation variable provided a holistic measure of maternal SES, that allowed for the categorization of the most advantaged and most disadvantaged groups within the study cohort. The HP index is also specific to the population of Ireland. The availability of maternal information and demographics from the original ROLO pregnancy study, in combination with the 5-year-old follow-up data, allowed for adjustment of important potential confounders in all analyses. All anthropometric measurements were measured by trained researchers. This study has a number of limitations. This study is cross-sectional in design, which precludes ability to infer causality. Selection bias may have been present as all mothers from the original ROLO pregnancy study were healthy and on their second pregnancy. Another limitation is that the majority of participants were in the higher SES group, with more than half of the original ROLO cohort having achieved a third-level education or more. Therefore, this sample may not be fully representative of the general population. Data provided by mothers on screen time exposure could have been strengthened if the questions on screen time had been repeated on several occasions and had included a request to mothers to keep an example diary. The CEBQ and the CLASS questionnaires have not been validated in an Irish population. Another limitation of this study is that the CEBQ is a parent reported questionnaire and therefore responses may be subject to social desirability bias. Although we adjusted for key confounders, there are other important potential confounders that were not included such as the child’s physical activity levels, parental feeding styles and parents eating behaviors. Controlling for these would have strengthened this research.

## Conclusions

This study adds insight into how three predictors, of childhood overweight, contained within the ecological framework; maternal SES, child screen time exposure and childcare arrangements, are associated with child eating behaviours in children aged 5 years old. The association between increased ‘Desire to Drink’ and lower SES may point to learned dietary patterns within the home environment. To our knowledge, our finding regarding the association between screen time exposure and food fussiness is novel, and requires further exploration to understand the direction of this association. Understanding how ecological factors, particularly in early childhood, impact a child’s eating behaviors is important in taking a systems approach to prevention of overweight and obesity*.*

## Supplementary Information

**Additional file 1: Table 1. **Maternal and child characteristics according to socio-economic status category at 5-year follow-up.

**Additional file 2: Table 2.** Breastfeeding exposure across maternal SES.

**Additional file 3: Table 3.** Correlation between duration of time child exposed to childcare and child eating behaviours at 5 years old.

## Data Availability

The datasets used and analyzed during the current study are not publicly available in line with ethical approval but are available from the corresponding author on reasonable request.

## Abbreviations

SES: Socio-economic status
ROLO: Randomized control trial of low glycemic index diet in pregnancy
CEBQ: Children’s Eating Behaviour Questionnaire
