# Supplementary material for: Ecological factors and childhood eating behaviours at 5 years of age: findings from the ROLO longitudinal birth cohort study
Source: BMC Pediatr. 2022 Jun 27;22:366. doi: 10.1186/s12887-022-03423-x (PMC9235107; doi:10.1186/s12887-022-03423-x)
Supplement: Supplementary file 1 — Additional file 1: Table 1. Maternal and child characteristics according to socio-economic status category at 5-year follow-up. [file 12887_2022_3423_MOESM1_ESM.docx]

| **Table 1****: Maternal and child characteristics according to socio-economic status category at 5-year follow-up** | | | | | | | | | | | | | | | | |
| --- | --- | --- | --- | --- | --- | --- | --- | --- | --- | --- | --- | --- | --- | --- | --- | --- |
|  | **Third level**  **and Advantaged** | | | | | **Third level**  **and Disadvantaged** | | | **Less than third level and Advantaged** | | | **Less than third level and Disadvantaged** | | |  | |
|  | **n** | | **Mean**  **(Median)** | | **SD**  **(IQR)** | **n** | **Mean**  **(Median)** | **SD**  **(IQR)** | **n** | **Mean**  **(Median)** | **SD**  **(IQR)** | **n** | **Mean**  **(Median)** | **SD**  **(IQR)** | **P-value** | |
| Maternal age 5-year f/up (years) | | 145 | | 39.42^a^ | 3.21 | 30 | 38.34^ab^ | 3.52 | 77 | 37.84^b^ | 3.98 | 28 | 35.83^c^ | 5.24 | <0.001 |  |
| Maternal BMI 5yr f/up (kg/m^2^)* | | 140 | | (24.35)^a^ | (22.42,26.85) | 27 | (25.46)^ab^ | (22.76,29.20) | 74 | (25.58)^b^ | (23.78,29.82) | 28 | (25.04)^b^ | (23.73,29.96) | 0.03 |  |
| Food Responsiveness (FR) | | 145 | | 2.45 | 0.71 | 30 | 2.54 | 0.94 | 77 | 2.61 | 0.84 | 28 | 2.48 | 1.08 | 0.34 |  |
| Emotional Overeating (EOE) | | 145 | | 1.67 | 0.54 | 30 | 1.71 | 0.53 | 77 | 1.62 | 0.41 | 28 | 1.61 | 0.63 | 0.79 |  |
| Enjoyment of Food (EF) | | 145 | | 3.73 | 0.58 | 30 | 3.80 | 0.58 | 77 | 3.65 | 0.83 | 28 | 3.71 | 0.96 | 0.75 |  |
| Desire to Drink (DD) | | 145 | | 2.51^a^ | 0.81 | 30 | 2.72^ab^ | 1.02 | 77 | 2.84^b^ | 0.98 | 28 | 3.01^b^ | 1.08 | 0.01 |  |
| Satiety Responsiveness (SR) | | 145 | | 3.07 | 0.61 | 30 | 2.82 | 0.56 | 77 | 3.10 | 0.72 | 28 | 3.09 | 0.73 | 0.21 |  |
| Slowness Eating (SE) | | 145 | | 3.04 | 0.77 | 30 | 2.76 | 0.68 | 77 | 3.08 | 0.78 | 28 | 3.31 | 0.85 | 0.05 |  |
| Emotional Undereating (EUE) | | 145 | | 2.75 | 0.87 | 30 | 2.83 | 0.84 | 77 | 2.65 | 0.83 | 28 | 2.65 | 0.99 | 0.73 |  |
| Food Fussiness (FF) | | 145 | | 3.10 | 0.97 | 30 | 3.11 | 0.94 | 77 | 3.28 | 0.94 | 28 | 2.74 | 1.20 | 0.11 |  |
| Age introduced to solids (weeks) | | 144 | | 23.87 | 7.30 | 27 | 23.54 | 7.29 | 91 | 21.90 | 5.54 | 26 | 20.73 | 5.86 | 0.05 |  |
| *Different letters denote differences between groups. p-value is for one-way ANOVA with post-hoc Tukey’s test;*Kruskal Wallis and Mann-Whitney U tests used for Maternal BMI, with median and IQR (25^th^-75th centile) presented for this variable. Food approach eating behaviours: degree to which a child has a more avid appetite and greater interest in food (includes FR, EOE,EF,DD), Food avoidant eating behaviours: degree to which a child has a smaller appetite and is less interested in food (includes SR, SE, EUE, FF). Abbreviations; IQR; Interquartile range; f/up- follow-up. Statistically significant (p value < 0.05)* | | | | | | | | | | | | | | | | |

**Additional file 1**
