# Supplementary material for: Ecological factors and childhood eating behaviours at 5 years of age: findings from the ROLO longitudinal birth cohort study
Source: BMC Pediatr. 2022 Jun 27;22:366. doi: 10.1186/s12887-022-03423-x (PMC9235107; doi:10.1186/s12887-022-03423-x)
Supplement: Supplementary file 2 — Additional file 2: Table 2. Breastfeeding exposure across maternal SES. [file 12887_2022_3423_MOESM2_ESM.docx]

| **Additional file 2**  **Table 2:** **Breastfeeding exposure across maternal SES** | | | | | |
| --- | --- | --- | --- | --- | --- |
|  | **Breastfed (yes) (df=3)** | | | | |
|  | **n** | **%** | **Pearsons X^2^** | **P-value** | **Phi** |
| Third level and Advantaged | 142 | 81.1 | 48.72 | <0.001 | 0.37 |
| Third level and Disadvantaged | 39 | 69.8 |  |  |  |
| Less than third level and Advantaged | 53 | 55.2 |  |  |  |
| Less than third level and Disadvantaged | 10 | 27 |  |  |  |
| *Chi squared test for independence* | | | | | |
