# Supplementary material for: Ecological factors and childhood eating behaviours at 5 years of age: findings from the ROLO longitudinal birth cohort study
Source: BMC Pediatr. 2022 Jun 27;22:366. doi: 10.1186/s12887-022-03423-x (PMC9235107; doi:10.1186/s12887-022-03423-x)
Supplement: Supplementary file 3 — Additional file 3: Table 3. Correlation between duration of time child exposed to childcare and child eating behaviours at 5 years old. [file 12887_2022_3423_MOESM3_ESM.docx]

| **Correlation between duration of time child exposed to childcare and child eating behaviours at 5 years old** | | | |
| --- | --- | --- | --- |
|  | **Childcare duration (years)** | | |
|  | **n** | **r** | **p-value** |
| Food Responsiveness (FR) | 232 | 0.087 | 0.188 |
| Emotional Overeating (EOE) | 232 | 0.102 | 0.122 |
| Enjoys Food (EF) | 232 | 0.033 | 0.616 |
| Desire to Drink (DD) | 232 | -0.076 | 0.249 |
| Satiety Responsiveness (SR) | 232 | -0.115 | 0.080 |
| Slowness Eating (SE) | 232 | -0.006 | 0.929 |
| Emotional Undereating (EU) | 232 | -0.089 | 0.075 |
| Food Fussiness (FF) | 232 | 0.094 | 0.155 |
| *Values generated from Spearman’s correlation statistic; Food approach eating behaviours: degree to which a child has a more avid appetite and greater interest in food (includes FR, EOE,EF,DD), Food avoidant eating behaviours: degree to which a child has a smaller appetite and is less interested in food (includes SR, SE, EUE, FF). Statistically significant (p-value <0.05)* | | | |

**Additional file 3**
